# The validity of an isiZulu speech reception threshold test for use with adult isiZulu speakers

**DOI:** 10.4102/sajcd.v67i1.690

**Published:** 2020-11-09

**Authors:** Seema Panday, Harsha Kathard, Wayne J. Wilson

**Affiliations:** 1 Department of Audiology, School of Health Sciences, University of Kwa Zulu-Natal, Durban, South Africa; 2 Department of Health and Rehabilitation Sciences, Faculty of Health Sciences, University of Cape Town, Cape Town, South Africa; 3 School of Health and Rehabilitation Sciences, Faculty of Health and Behavioural Sciences, The University of Queensland, Queensland, Australia

**Keywords:** speech reception threshold, convergent validity, concurrent validity, adults, isiZulu

## Abstract

**Background:**

This study continued the development of an isiZulu speech reception threshold (zSRT) test for use with first language, adult speakers of isiZulu.

**Objectives:**

The objective of this study was to determine the convergent and concurrent validity of the zSRT test.

**Methods:**

One hundred adult isiZulu first-language speakers with normal hearing and 76 first-language, adult isiZulu speakers with conductive or sensorineural hearing losses ranging from mild to severe were assessed on pure tone audiometry and a newly developed isiZulu SRT test. Convergent validity was established through agreement of the zSRT scores with pure tone average (PTA) scores. Concurrent validity was assessed by examining the steepness of the psychometric curve for each word in the zSRT test for each type and degree of hearing loss.

**Results:**

Intraclass correlation coefficient analyses showed zSRT scores were in substantial to very high agreement with PTA scores for the normal hearing and hearing loss groups (NH – right ear ICC consistency = 0.78, left ear ICC = 0.67; HL – right ear ICC consistency = 0.97, left ear ICC consistency = 0.95). The mean psychometric slope (%/dB) at 50% correct perception for all words in the zSRT test was 4.92%/dB for the mild conductive hearing loss group, 5.26%/dB for the moderate conductive hearing loss group, 2.85%/dB for the moderately severe sensorineural hearing loss group and 2.47%/dB for the severe sensorineural hearing loss group. These slopes were appropriate for the degree of hearing loss observed in each group.

**Conclusion:**

The zSRT test showed convergent and concurrent validity for assessing SRT in first language, adult speakers of isiZulu.

## Introduction

This article reports on the convergent and concurrent validity of an isiZulu speech reception threshold (zSRT) test for use with first-language, adult speakers of isiZulu with and without hearing loss.

With evidence-based practice now being prioritised for clinical practice, the need for validity evidence of newly developed tests continues to grow amongst clinicians and researchers alike (Friberg & McNamara, 2010). In the case of speech audiometry, evidence of the validity of its tests has been dominated by reports of the psychometric functions (also called the performance intensity functions) for the word stimuli used in these speech audiometry tests (e.g., Harris et al., 2001; 2004; Harris, Kim, & Egget, 2003; Nissen, Harris, Jennings, Eggett, & Buck, 2005; Nissen, Harris, & Slade, 2007). Whilst such reports provide some evidence of validity and reliability, systematic assessments of the validity of speech audiometry tests remain rare. This is concerning as the clinical use of any test in audiology should be based on a rigorous, strict and systematic investigation of its validity and reliability, and the lack of such investigation limits the use of these tests in clinical practice (Ma, McPherson, & Ma, 2013).

In general, validity refers to the degree to which a test or measurement tool achieves what it is supposed to measure (Maxwell & Satake, 2006; Mendel, 2008). Historically, validity has been presented as separate subtypes (Cronbrach & Meehl, 1995) including construct validity – the degree to which a test or instrument measures the theoretical construct under investigation. It is recommended that all subtypes of validity be considered for test validation, with authors such as Messick (1995) offering unified validation frameworks to achieve this goal. Central to this framework’s consideration of validity is the placement of construct validity as the overarching type of validity (Messick, 1995, in Dellinger & Nancy, 2007). Construct validity is then broken down into content, convergent and divergent, and criterion or concurrent validity. Messick’s (1995) framework is useful as it allows several levels of evidence to be gathered to argue for or against the overall validity of any new test. For this article evidence relating to convergent and concurrent validity will be the focus.

Speech reception threshold (SRT) testing remains a core component of the audiological test battery (Ramkissoon, Estis, & Flagge, 2014). On hearing a list of words that have been pre-recorded or presented by live voice, the listener is asked to repeat each word as heard. By scoring the responses, an examiner can determine the listener’s SRT as the level at which he or she correctly repeated the words 50% of the time. This SRT score can then be used to quantify the listener’s speech reception ability, to cross-check other audiometric results such as the pure tone average threshold obtained from pure tone audiometry, to confirm or deny sites of lesion along the auditory pathway and to guide some forms of auditory rehabilitation (Gelfand, 2001).

Whilst SRT testing has been used in audiology clinics around the world since the 1950s, it continues to face several challenges. Perhaps the largest of these challenges is the need to match the words used in an SRT test to the contextual, linguistic and clinical factors relevant to the target population. Such a challenge is of particular relevance in a country such as South Africa that has 11 official languages: Afrikaans, English, Ndebele, Northern Sotho, Sotho, Swazi, Tsonga, Tswana, Venda, Xhosa and Zulu.

At present, the majority of SRT tests have been developed in the English language and particularly in American English (Ballachanda, 2001; Ramkissoon, Proctor, Lansing & Bilger, 2002). Use of these tests in South Africa has some (albeit limited) prima facie value with South African English being spoken as the primary language at home by 8.1% of the population (Statistics South Africa, 2018), making it the sixth most commonly primary spoken language in South Africa after isiZulu (23%), isiXhosa (16%) and Afrikaans (14%). South African English is also one of several languages commonly used in most urban areas in South Africa (Statistics South Africa, 2018) and is the dominant language spoken in government and media settings. Despite this, it remains clear that the use of SRT tests in English (and the dominant American English) on the South African population is culturally and linguistically inappropriate at best (Ramkissoon et al., 2002) and invalid at worst (Aleksandrovsky, McCullough, & Wilson, 1998; Martin & Hart, 1978; Harris et al., 2003, 2004; Ramkissoon et al., 2002).

In response to the need for valid speech audiometry tests in South Africa, researchers have begun to develop locally relevant tests (Hanekom, Soer, & Pottas, 2015; Khoza, Ramma, Mophosho, & Moroka, 2008; Naude, 2018). This includes the development of an SRT test in the most spoken language in South Africa, isiZulu, which is spoken as the primary language at home by approximately 23% of the population (Statistics South Africa, 2018; Panday, Kathard, Pillay, & Govender, 2007, 2009; Panday, Kathard, Pillay, & Wilson, 2018a, 2018b). The current recording of this zSRT test consists of 28 common, bisyllabic isiZulu words spoken by a male, first-language speaker of isiZulu. These words have been shown to be linguistically familiar and homogenous in audibility in first-language, adult speakers of isiZulu (Panday et al., 2007, 2009, 2018a, 2018b) and this test has been shown to produce reliable SRTs when used on such adults with normal hearing sensitivity (Panday et al., 2018a). If this new zSRT test is to be used on first-language, adult speakers of isiZulu in South Africa, then its validity when applied to individuals with and without hearing loss must be assessed.

The aim of this study was to determine whether the zSRT test is a valid measure of SRT in the first- language, adult speakers of isiZulu with and without hearing loss.

## Methods

### Research design

This study used an analytical, observational design (Maxwell & Satake, 2006) to collect and analyse quantitative data in two parts, one involving participants with normal hearing and one involving participants with hearing loss.

### Participants

All participants were first-language, adult speakers of isiZulu (self-reported) and permanent residents of KwaZulu-Natal.

For part one of the present study, 100 participants with normal hearing aged 18–60 years (mean age 37 years) were recruited through advertisements and selected based on consecutive sampling (Maxwell & Satake, 2006) from the eThekwini and surrounding areas of Kwa Zulu-Natal. Participants had to have unremarkable case history and their hearing status had to be confirmed by audiometric testing.

For part two of the present study, 76 adults with hearing loss between the ages of 21 and 59 years (mean age 39 years) were purposely sampled from the clinical databases of two audiology departments in two provincial hospitals and the audiology clinic of a university in EThekwini, Kwa Zulu-Natal, South Africa. The hearing status of each participant was identified from their hospital or clinical database records and confirmed by audiometric assessment at the research facility. These 76 participants were grouped by the degree of hearing loss in their better hearing ear. This created four groups: mild conductive hearing loss (26–40 dB, *n* = 15), moderate conductive hearing loss (41–55 dB, *n* = 20), moderately severe sensorineural hearing loss (56–70 dB, *n* = 21) and severe sensorineural hearing loss (71–90 dB, *n* = 20).

### The isiZulu speech reception threshold test

The zSRT test used in this study has been reported in detail by Panday et al. (2007, 2009, 2018a, 2018b). It consists of 28 bisyllabic isiZulu low-tone verbs that are linguistically familiar and homogenous in audibility for isiZulu-speaking adults. The words were recorded to a compact disk with a calibration tone and test instructions (in isiZulu) for the listener.

### Test administrator

All tests were conducted by an audiologist with 6 years clinical experience who had been trained by the present study’s first author (Panday). This audiologist was the first-language speaker of isiZulu.

### Procedure

All tests were performed in an isolated Industrial Acoustics Company twin audiometric soundproof booth of double wall construction meeting ANSI (1977) standards using a Grayson-Stradler GSI 61 twin channel clinical audiometer with TDH-49 Telephonics earphones and MX41-AR cushions, a Technics (SLPG390) compact disk player and a GSI Tympstar clinical middle ear analyser.

Testing occurred in two sessions. Participants in part one of the present study only participated in testing session one. Participants in part two of the present study participated in testing sessions one and two.

### Session one

In the first session, all participants completed pure-tone audiometry, tympanometry and SRT testing using the zSRT test. Pure tone audiometry was conducted at octave frequencies from 250 Hz to 8000 Hz using the modified Hughson-Westlake threshold technique (Carhart & Jerger, 1959). The pure tone average (PTA) (the average hearing threshold for 0.5, 1 and 2 kHz) and the zSRT were calculated and recorded.

For the zSRT testing, each participant listened to the words being read aloud by the tester, and was given the opportunity to clarify any unfamiliar words (as recommended by ASHA, 1988). A modified version of the Chaiklin and Ventry (1964) descending method (as cited in Gelfand, 2001) was used to conduct the SRT testing. This method was modified by changing the starting level from 25 dB SL relative to a two frequency pure-tone average to 10 dB SL relative to the three frequency (0.5, 1 and 2 kHz) pure-tone average. If the participant was unable to repeat the initial word played, then the SRT test sequence was restarted by presenting a new word at the initial presentation level plus 10 dB. Once the participant was able to repeat a word, then a further two words were presented at the same presentation level. If all three words were not correctly repeated, the audiologist increased the presentation level by 5 dB and played the next three words. Once all three words were correctly repeated, the audiologist reduced the presentation level by 5 dB and played the next three words. This was repeated until the participant incorrectly repeated any one of the words in a three-word block, at which point the audiologist played another three words at the same level to get a score out of six. If the participant correctly repeated three out of the six words, then the presentation level of those words was deemed to be the participant’s SRT and the SRT testing was stopped. If the participant correctly repeated fewer than three out of the six words, then the presentation level was increased by 5 dB, and another set of six words were presented. If the participant correctly repeated more than three out of the six words, then the presentation level was decreased by 5 dB and another set of six words were presented. This sequence of increasing or decreasing the presentation level was repeated until the participant correctly repeated three out of a six word block, the presentation level of which was deemed to be the participant’s SRT and the SRT testing was stopped. To brief, a participant’s SRT was deemed to be the lowest presentation level at which he or she correctly repeated three out of a block of six words.

### Session two

In the second session, only participants with hearing loss completed word recognition testing using the zSRT test words played at varying presentation levels to obtain psychometric functions for each word in the zSRT test. Each participant was instructed in isiZulu by the test administrator that a series of words would be played through the headphones to his or her better hearing (or to the right ear if the hearing was symmetrical). The task was to repeat each word as heard.

All 28 words on the zSRT test were first played at 20 dB SL to familiarise each participant with the words, and then in a randomised order at 35–70 dB HL (dial setting) in 5 dB steps for participants with mild conductive hearing loss, at 40–75 dB HL (dial setting) in 5 dB steps for participants with moderate conductive hearing loss, 50–85 dB HL (dial setting) in 5 dB steps for participants with moderately severe sensorineural hearing loss and at 70–95 dB HL (dial setting) in 5 dB steps for participants with severe sensorineural hearing loss. Participants were given two 5-min rest breaks during this testing to improve co-operation and reduce fatigue. All participant responses were scored by two scorers (the researcher and the isiZulu-speaking audiologists).

### Data analysis

The PTA (the average hearing threshold for 0.5, 1 and 2 kHz) and zSRT scores for both participant groups were confirmed as meeting parametric assumptions by inspecting their histograms, box and whisker plots, and Q-Q plots (data not shown). Agreement between the PTA and zSRT results for each participant group separately, for right and left ears separately, were assessed using intra-class correlation coefficient (ICC) analyses (Bartlett & Frost, 2008; Rankin & Stokes, 1998; Shrout & Fleiss, 1979). The strength of these ICC values was classified using the general (although arbitrary) guidelines reported by Landis and Koch (1977) of < 0 indicating poor agreement, 0.01–0.20 indicating slight agreement, 0.21–0.40 indicating fair agreement, 0.41–0.60 indicating moderate agreement, 0.61–0.80 indicating substantial agreement and 0.81–1.00 indicating almost perfect agreement. Differences between the PTA and zSRT results for all participants as a single group, for right and left ears separately, were assessed using linear mixed model analyses. These analyses were conducted with threshold (dB HL) as the dependent variable, threshold method (PTA, zSRT) as an independent variable and fixed effect, participants as an independent variable and random effect (with unstructured co-variance type) and the intercept included in the model.

From session two, logistic regression analyses were used to determine the psychometric function of each of the 28 words in the zSRT recording for each of the four subgroups in the hearing loss participant group separately. For each word in the zSRT recording, and for each participant subgroup, logistic regression equations were fitted, the slope of each model was determined and the word threshold (50% correct) was calculated. This analysis was not completed for the normal hearing participant group as it had been completed on a separate sample of participants with normal hearing and reported elsewhere (Panday et al., 2009, 2018a).

All statistics were conducted using IBM SPSS Statistics, version 24, release 24.0.0.0 for personal computers.

### Ethical consideration

Ethical clearance was granted by the Faculty of Health Sciences Human Research Ethics Committee of the University of Cape Town to conduct the study (clearance number: HREC 652/2012) and subsequent annual clearance was obtained during the data collection period. Informed consent was obtained from all participants prior to their participation in the study.

## Results

For part one of the present study, Table 1 shows the mean PTA and SRT values for participants with normal hearing and with hearing loss. Table 2 shows the intraclass correlation coefficients (ICC) between the two threshold measures (PTA and zSRT) for each group by ear. The ICC values ranged between 0.67 and 0.88 for each ear for both single measures (single threshold method) and average measures (averaged between both threshold methods) for the normal hearing group and were > 0.95 for single measures (single threshold method) and average measures (averaged between both threshold methods) for the hearing loss group.

**TABLE 1:** Pure tone average and isiZulu speech reception threshold measurements for right and left ears for participants with normal hearing and with hearing loss.

Variable	Pure tone average (dB HL)		Speech reception threshold (dB HL)	
	Right ear	Left ear	Right ear	Left ear
**Normal hearing group** (*n* = 100)				
Mean	8.20	7.41	11.25	10.70
SD	5.48	5.57	5.28	5.45
**Hearing loss group** (*n* = 76)				
Mean	56.53	58.53	58.42	57.37
SD	21.72	23.19	22.63	21.25

HL, hearing loss; SD, standard deviation.

**TABLE 2:** Intraclass correlation coefficient (2, 1) results for agreement between pure tone average and isiZulu speech reception threshold measures for participants with normal hearing and with hearing loss.

Variable	ICC coefficient	95% CI	*F*-test value	*df*
**ICC (2,1) for normal hearing group** (*n* = 100)				
**RE (NH)**				
Single measure	0.78	0.69–0.84	99.0	75
Average measure	0.88	0.81–0.91	99.0	75
**LE (NH)**				
Single measure	0.67	0.55–0.77	99.0	75
Average measure	0.80	0.71–0.87	99.0	75
**ICC(2,1) for hearing loss group** (*n* =76)				
**RE (HL)**				
Single measure	0.97	0.95–0.98	86.31	75
Average measure	0.99	0.98–0.99	86.31	75
**LE (HL)**				
Single measure	0.95	0.90–0.97	86.31	75
Average measure	0.98	0.96–0.99	86.31	75

ICC, intraclass correlation coefficient; HL, hearing loss; LE, left ear; NH, normal hearing; RE, right ear.

Note: Single measure refers to each threshold measure on its own and the average measure refers to average between the two threshold measures.

Table 3 shows results of the linear mixed model analyses for the both participant groups by ear for differences between the two threshold measures of PTA and zSRT. A significant (*p* < 0.001) difference between PTA and zSRT was observed for each participant group.

**TABLE 3:** The linear mixed model analysis results for differences in thresholds by threshold measure (pure tone average vs. isiZulu speech reception threshold) for participants with normal hearing and participants with hearing loss.

Ear	Parameter	Estimate	Standard error	*t*	Sig	95% confidence interval
**Normal hearing group** (*n* =100)						
Right ear	Intercept	11.10	1.57	7.06	< 0.001	8.02 – 14.18
	PTA	−3.08	0.75	−4.10	< 0.001	−4.575 – -1.60
	SRT	0	-	-	-	-
Left ear	Intercept	10.40	4.03	2.57	< 0.001	1.28 – 19.52
	PTA	−2.91	0.79	−3.65	< 0.001	−4.49 – -1.34
	SRT	0	-	-	-	-
**Hearing loss group** (*n* = 76)						
Right ear	Intercept	60.87	2.50	24.34	< 0.001	55.90 – 65.83
	PTA	−2.04	0.64	−3.18	0.002	−3.32 – -0.77
	SRT	0	-	-	-	-
Left ear	Intercept	61.14	2.58	23.74	< 0.001	50.02 – 66.26
	PTA	−3.03	0.64	−4.77	< 0.001	−4.30 – -1.77
	SRT	0	-	-	-	-

PTA, pure tone average; SRT, speech reception threshold.

**FIGURE 1:**
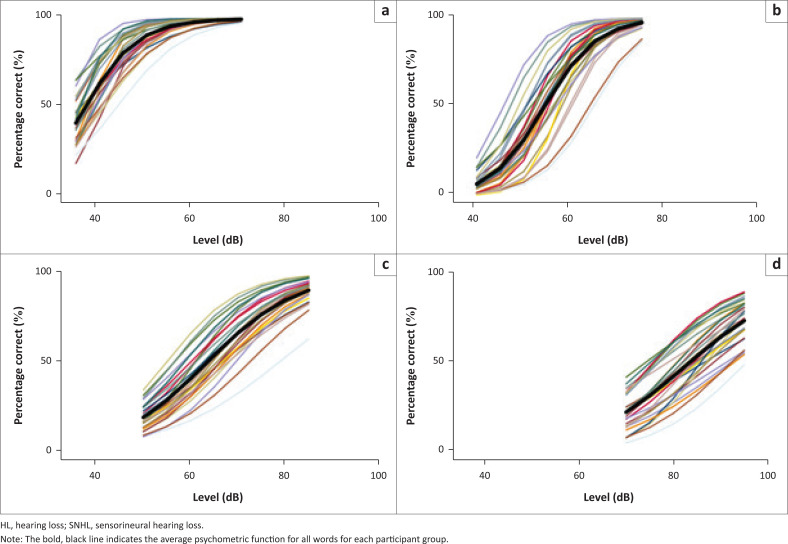
The psychometric functions for each of the 28 words in the zSRT speech reception threshold test, for each participant group by degree of hearing loss: (a) mild conductive hearing loss (b) moderate conductive hearing loss (c) moderately severe SNHL (d) Severe SNHL.

For part two of the present study, Table 4 shows the psychometric function measures and Figure 1 shows the psychometric functions for the participants with hearing loss by degree of loss. The psychometric data obtained from the present study’s normally hearing participants are not reported here as they have already been reported in Panday et al. (2018a). Psychometric data obtained from another group of normally hearing participants using a precursor zSRT recording to the one used in the present study have also been reported by Panday et al. (2009).

**TABLE 4:** Psychometric function measures for the 28 isiZulu words for the participants by degree of hearing loss.

Variable	Mild conductive hearing loss			Moderate conductive hearing loss			Moderately to severe sensorineural hearing loss			Severe sensorineural hearing loss		
	Slope	Slope at 20%–80% correct	Threshold	Slope	Slope at 20%–80% correct	Threshold	Slope	Slope at 20%–80% correct	Threshold	Slope	Slope 20%–80% correct	Threshold
Mean	4.92	3.15	37.09	5.26	3.37	54.46	2.85	1.82	64.15	2.47	1.58	82.68
SD	1.26	0.81	3.04	0.95	0.61	4.40	0.41	0.26	5.21	0.53	0.34	5.93
Minimum	2.88	1.84	30.40	3.65	2.34	45.59	2.31	1.36	55.11	1.63	1.04	73.09
Maximum	7.63	4.88	43.64	7.63	4.88	64.68	3.75	2.40	79.00	3.68	2.35	94.68
Range	4.75	3.04	13.24	3.98	2.54	19.09	1.63	1.04	23.89	2.05	1.31	21.59

SD, standard deviation.

## Discussion

The present study’s results suggest that the zSRT test is a valid measure of SRT in first language, adult speakers of isiZulu with and without hearing loss.

The convergent validity of the zSRT test was supported by the very high agreement (ICC values ≥ 0.80) observed between participant PTA and zSRT values for participants with and without hearing loss, for both right and left ears. This support is drawn from this study’s use of the zSRT and PTA scores as measures of the theoretical construct of hearing threshold. The degree to which the zSRT and PTA measures of this construct were related suggests high convergent validity. The zSRT results can reasonably be expected to show high levels of agreement with the established PTA results for first-language, adult speakers of isiZulu with hearing loss, when these tests are applied in clinical settings.

Whilst the zSRT and PTA values were in very high agreement in this study’s participants, mixed model analyses showed the zSRT values to be significantly (*p* < 0.005) higher than the PTA values by an average of 2.9–3.0 dB for participants with normal hearing and by an average of 2–3 dB for participants with hearing loss. Whilst statistically significant, these differences were not considered to be clinically significant given the minimum step size for presentation level used in this study was 5 dB, and differences 6 dB or less between SRT and PTA having been taken to indicate agreement between those two measures for tests in several languages (Carhart, 1971; Han et al., 2011; Marinova-Todd, Siu, & Jensad, 2011; Sreedhar, Ventatesh, Nagaraja, & Srinavasan, 2011; Wang, Mannel, Newall, Zhang, & Han, 2007).

The concurrent validity of the zSRT test was supported by the psychometric functions for each word in the zSRT test being consistent with the degrees and types of hearing loss shown by the study’s participants with hearing loss by subgroup, that is, mild conductive, moderate conductive, moderately severe sensorineural and severe sensorineural hearing loss. For increasing degrees of hearing loss, the psychometric functions showed the expected increases in threshold and decreases in slopes (Wilson & Carter, 2001) for the zSRT test words. Similarly, Wang et al. (2007) found that when participants with sensorineural hearing loss were evaluated with Mandarin bisyllabic words, the psychometric functions slopes were more shallow compared with results with normal hearing counterparts.

A closer inspection of the psychometric functions of individual words in the isiZulu test across the four hearing loss groups indicates some variability in curves amongst individual words. For example, the psychometric functions for the words /wina/ and /minya/ were consistently very shallow for all degrees and types of hearing loss. This suggests these words could be lowering the concurrent validity of the zSRT test overall and the removal of these words from the zSRT test wordlist could be considered.

### Clinical implications

The present study’s findings further support the potential use of the zSRT test as a valid and reliable measure of SRT in first-language, adult speakers of isiZulu speakers. This zSRT test has now been shown to contain words that are linguistically familiar and homogenous in audibility in isiZulu-speaking adults (Panday et al., 2007, 2009, 2018a, 2018b), to produce reliable SRTs when used on adult speakers of isiZulu with normal hearing sensitivity (Panday et al., 2018b), and to produce valid SRTs when used on adult speakers of isiZulu with and without hearing loss (current study).

The present and previous studies reporting the development of this zSRT test also support the use of rigorous, systematic and multiple methods particularly based on a unified validation framework to accumulate evidence for the validity and reliability of any new test. Previous attempts to use only one or two methods to confirm the validity and/or reliability of new tests should not be considered sufficient for such purposes. It is important to view multiple sources of evidence regarding the validity and reliability on new tests before such tests can be properly considered for clinical use.

## Conclusion

The present study’s results suggest the zSRT test is a valid measure of SRT in first-language, adult speakers of isiZulu with and without hearing loss, showing both convergent and concurrent validity when used in these populations.

This study’s findings are limited by its participants being predominantly recruited from eThekwini and surrounding regions in KwaZulu-Natal, South Africa, although the test words did represent a central dialect of isiZulu. It could also be limited by its use of the SRT method described by Chaiklin and Ventry (1964, cited in Gelfand (2001) with modification, which may not immediately generalise to SRT scores contained using other methods.
